# Michael Balint's Word Trail: The ‘Ocnophil’, the ‘Philobat’ and Creative Dyads

**DOI:** 10.3366/pah.2019.0281

**Published:** 2019-04

**Authors:** Raluca Soreanu

**Affiliations:** ^A^ RALUCA SOREANU, PhD, is Wellcome Trust Fellow in Medical Humanities at the Department of Psychosocial Studies, Birkbeck College, London and psychoanalyst, effective member of Círculo Psicanalítico do Rio de Janeiro. She holds a PhD in Sociology, from University College London, and is the author of *Working*-*through Collective Wounds: Trauma, Denial, Recognition in the Brazilian Uprising* (Palgrave, 2018). Address for correspondence: Raluca Soreanu, PhD, Department of Psychosocial Studies, School of Social Science, History and Philosophy, Birkbeck, University of London, 26 Russell Square, Room 230, Bloomsbury, London, WC1B 5DQ, UK.

**Keywords:** Michael Balint, David Eichholz, ocnophil, philobat, Budapest School of psychoanalysis

## Abstract

In this paper, I discuss how Michael Balint arrived at the concepts of ‘ocnophil’ and ‘philobat’, which refer to two kinds of object relations. I look at the correspondence between Balint and the classical scholar David Eichholz. The two crafted these words together in a passionate exchange of letters. By recognizing the importance of creative dyads in psychoanalysis, we gain more insight into the creation of psychoanalytic knowledge beyond the frame of individual authorship. I read the collaboration between Balint and Eichholz in its historical and theoretical context, particularly in relation to the Budapest School of psychoanalysis, where intellectual collaborations had an important place. The Budapest School was Michael Balint's first home, and it shaped his epistemic and psychoanalytic style. Balint constructed his psychoanalytic theories in a spirit of openness, maintaining a commitment to conversations between psychoanalysis and other disciplines.

## From Authors to Dyads: Untold Histories of Psychoanalysis

In organizing and passing on psychoanalytic knowledge, our most enduring epistemic ‘units’ are individual authors and schools. As Michel Foucault famously wrote, ‘[t]he coming into being of the notion of “author” constitutes the privileged moment of individualization in the history of ideas, knowledge, literature, philosophy, and the sciences’ (Foucault, 1984[1977], p. 133). In the following pages, I start from an exchange of letters between psychoanalyst Michael Balint and classical scholar David Eichholz, so as to reflect on what we miss in making sense of psychoanalytic knowledge production by grounding ourselves in authors and schools, and on what we might gain from discovering other forms of authorship, such as the dyad. I argue that the event of crystallizing an individual auctorial voice from multiple exchanges and conversations is not a benign one. A psychoanalytic reading of the disappearance or disavowal of certain voices in the field is just as important as imagining new non-individual forms of authorship. In discussing the unrecognized contributions of Sabina Spielrein to psychoanalytic theory, Adrienne Harris asks, ‘Is this a story about the fate of women or outliers more generally in psychoanalysis, the propensity for eclipse and erasure that “disappeared” a number of figures, Ferenczi perhaps most significantly?’ (Harris, 2015, p. 728). My pursuit here is not to look at the forgotten individual authors, but at smaller or greater collaborations, at generative dyads.

In their essay ‘Why Psychoanalysis Has No History’, Elisabeth Young-Bruehl and Murray Schwartz comment on a regression of psychoanalytic history-writing into biographic writing, memorializing, or criticizing Freud. What is creating this proliferation of fragmented stories is the trauma history of psychoanalysis, which remains largely unacknowledged. The trauma relates in important ways to the migration of psychoanalysts before and during World War II, mostly to England and to the Americas, and to its deep consequences in terms of dislocation and communal fragmentation (Young-Bruehl & Schwartz, 2012, p. 140). Here, Freud's dislocation and his death in England have a central place. What is missing, for Young-Bruehl and Schwartz, is a collective historical consciousness that can organize a set of disparate observations precisely as a trauma history, a reflection on ‘a repetitive pattern of splits and consequent distortions’ (Young-Bruehl & Schwartz, 2012, p. 142).^1^

While I resonate with Young-Bruehl and Schwartz in their insistence on tackling the traumatic residues resulting both from historical events and from intellectual splits, quarrels and fragmentations internal to the fields of psychoanalysis, I aim to stress the importance of ‘lateral’ histories in the making of psychoanalytic knowledge. It is crucial to look at great splits that arrested the psychoanalytic imagination – such as, for instance, the split between Freud and Ferenczi, which, as various authors have shown (Bergmann, 1996; Brabant, 2003; Haynal, 1997, 2002; Martín-Cabré, 1997; Schneider, 1988), has had traumatic consequences and has led to a decades-long forgetfulness around Ferenczi's contributions to psychoanalytic theory and technique.^2^ But it is equally important to find traces of ‘smaller’ generative collaborations between psychoanalysts and non-psychoanalysts. These ‘lateral’ histories are equally impregnated by the traumatic events mentioned above; but they also allow us to observe some form of working-through of these traumas. It is through these ‘lateral’ histories, which cut across fields of knowledge, that we can discern how the boundaries of psychoanalysis are made, un-made, and re-made.

The Balint–Eichholz correspondence counts as such a lateral history. It enables us to see Balint's epistemic style at work, in the act of construing two concepts that are at the core of his own theory of object relationality: ocnophil and philobat. It enables us to place him in the theoretical and epistemological ‘scene’ of the Budapest School of psychoanalysis; and in the historical context of dislocations around World War II. Finally, it enables us to think with Balint beyond Balint, beyond the form of ‘individual author’; and it opens a refreshing window onto a different generative form, the dyad – in this case, the Balint–Eichholz dyad, as it comes across through a series of letters, exchanged between August 1953 and February 1954.^3^ This dyad is not in any sense central to the development of contemporary psychoanalytic theory, but it is nevertheless an event of creativity that tells a story about the construction of psychoanalytic knowledge.

In what follows, I propose two ‘frames’ for the Balint–Eichholz correspondence and for understanding its broader significance. The first frame is a discussion of the practices of the Budapest School of psychoanalysis, focusing on Balint's collaborations and creations, and on the particular kind of multi-relationality that characterized his formative years. The second frame is a linguistic portrait of Balint, which illuminates why Balint was able to sustain the creative dyad that led to an adventure into the Greek language, and to the naming of the ocnophil and the philobat. As we will see, Balint lures Eichholz^4^ into the adventure of naming psychic states, in an act of faith that the psychoanalytic objects at hand are transmissible to other disciplines, that they can be understood by non-psychoanalysts, and that they can acquire a name through a conversation that happens across fields of knowledge and across languages. A fascinating detail of this correspondence is that the two distinct conceptions of language of the two men clash creatively, with Eichholz being puzzled by Balint's associationist and non-arbitrary theory of language.

## The Budapest School and Intellectual Collaborations

Michael Balint's psychoanalytic formative home was Budapest, although his official psychoanalytic formation took place during his years of exile in Berlin, between 1921 and 1924, at a time when the political climate in Hungary was becoming more and more difficult for its Jewish population. The Berlin Polyclinic, directed by Ernst Simmel, Max Eitingon and Karl Abraham, had been established shortly before Balint's arrival. Balint was among the first few to ‘test’ the Berlin training system, which he later on described as a defensive reaction against the years of little structure that preceded it (Balint, 1948, 1954).

In Budapest, the psychoanalytic beginnings were marked by a uniquely robust and effervescent pluridisciplinarity. In the first two decades of the twentieth century, the exchanges of avant-garde intellectuals (writers, musicians, painters, psychoanalysts, medical doctors, lawyers, economists) were organized in a number of forums (Mészáros, 2010, 2014). The medical weekly *Gyógyászat* [*Therapeutics*] had an important role in popularizing psychoanalytic ideas. Some of the main journals of literary criticism – such as *Nyugat* [*The West*] – as well as sociological ones – such as *Huszadik Század* [*The Twentieth Century*] – also played a crucial part in articulating psychoanalytic concerns. A group set up by students of medicine and engineering, A Galilei Kör [The Galileo Circle], openly pursued the goal of making psychoanalysis part of the university curriculum for training medical doctors. In the summer of 1919 Sándor Ferenczi was appointed professor in psychoanalysis, in the first department of psychoanalysis within a medical university (Mészáros, 2010; Erős *et al.*, 1987; Erős, this issue). While this appointment was short-lived, and it was revoked after only one month in the heat of the political events in Hungary, it did reflect the presence of psychoanalysis in Hungarian cultural life. Ferenczi was lecturing to full amphitheatres and to an enthusiastic audience. The voices of psychoanalysts were also heard in the national press, as they were often consulted on a great variety of topics, from psychopathology to matters of everyday life. Finally, many of the prominent literary figures of the time (such as, for instance, Sándor Márai) found inspiration in psychoanalytic ideas and constructed a psychoanalytically dense literary universe.

It is in this vibrant context of exchanges around psychoanalytic topics that Balint met Alice Székely-Kovács, his future wife and intellectual partner. While Michael had dedicated his interest to medicine and chemistry, Alice had a keen interest in both anthropology and psychoanalysis. In 1917, it is Alice who lends him *Totem and Taboo*, one of Balint's introductions to psychoanalysis, alongside the *Three Essays in the Theory of Sexuality*. In 1919, Balint also heard Ferenczi's university lectures. In the preface to *Primary Love and Psycho-Analytic Technique*, published in 1953, Balint gives us a description of the extent of his intellectual collaboration with Alice, and of the kind of psychoanalytic dyad they were part of:

Starting with our shared enthusiasm for *Totem and Taboo* till her death in 1939, Alice and I read, studied, lived, and worked together. All our ideas – no matter in whose mind they had first arisen – were enjoyed and then tested, probed and criticised in our endless discussions. Quite often it was just chance that decided which of us should publish a particular idea. (Balint, 1953, p. 6)

From an early point in his engagement with psychoanalysis, Michael Balint was thus inclined to become involved in intense dyadic exchanges, whose modes of creativity are readable only while thinking outside the form of the individual author. There is certainly an Alice–Michael dyad that is worth investigating.

What is notable about Balint's period in Berlin is that, apart from beginning his psychoanalytic training and pursuing a doctorate in natural sciences, he had the initiative, in 1922 and 1923, of experimenting with the psychotherapy of patients affected by organic diseases. He saw patients suffering from asthma, peptic ulcer, thyrotoxicosis and obesity. This experiment took place at the famous Medical Clinic of the Charité, with the approval of Professors His and Zondek (Balint, 1970). On the basis of this experience, he published the article ‘Psychoanalyse und klinische Medizin’ (Balint, 1926). Balint's epistemic disposition was that of enlarging the scope of psychoanalysis and ‘applying’ it to areas where it meets the medical sciences. It is with these early pursuits that he established himself as one of the pioneers of psychosomatic medicine. His later work with medical doctors, formalized as ‘Balint groups’, also started here, in the negotiations with doctors for giving a space to psychoanalysis in their clinical practice.

Upon his return to Budapest, in 1924, he initially encountered difficulties in obtaining support for continuing his project of psychoanalysis in hospitals, with patients suffering from organic illnesses (Balint, 1970). But another idea took shape, and occupied the minds and hearts of the psychoanalysts in Budapest: the opening of a psychoanalytic clinic. Ferenczi had been hoping for such a clinic since 1915. It is crucial to say that the Budapest Polyclinic – which opened its doors in December 1931, after years of struggle in the dire political times of Horthy's regime – had the same address as the couple Michael–Alice Balint: Mészáros utca 12. The clinic was hosted in a space on the ground floor of the large building, designed, built and owned by Michael's father-in-law, and Alice's stepfather, Frigyes Kovács. Frigyes was a successful architect, and the second husband of Vilma Kovács, one of the most prolific figures of Hungarian psychoanalysis, who left us key contributions on psychoanalytic training (Kovács, 1936). In an interview, Balint evokes the difficulties around creating this new institution:

I got the permission, after a long struggle with the authorities. Everyone was against it, of course, the medical profession, the university, the General Medical Council. Eventually we got the permission and we opened, and we had a very nice institute, with quite a good load of work. (Swerdloff, 2002, p. 391)

Even before the opening of the clinic, Mészáros u. 12 was a well-known meeting place for psychoanalysts, writers and musicians, friends of the Kovács family. With the clinic, Friday meetings became regular, and they brought together Sándor Ferenczi, Alice and Michael Balint, Vilma Kovács, and also Endre Almássy, Robert Bak, Lilly Hajdu, Imre Hermann, István Hollós, Kata Lévy, Edit Ludowyk-Gyömröi, Sigmund Pfeiffer, Géza Róheim and Lilian Rotter. Senior analysts gave lectures, and they were followed by a seminar in psychoanalytic technique, led by Vilma Kovács.

In the political landscape of interwar Hungary, the Polyclinic remained on the margin, in tension with the university and the medical establishment, and under political scrutiny. By 1937, when Balint was directing the clinic, a policeman in civilian clothes started attending their meetings and taking notes of everything that was said.

The Budapest Polyclinic stood apart from other early psychoanalytic clinics in that it regarded therapy as its primary mission, with training coming second. This order of priorities was discussed at length by its members, but it passed the test of collective agreement. We could say that the Polyclinic had a substantial autonomy from the Society: it was a fully-fledged therapeutic and training establishment. Balint got involved energetically in securing this state of affairs. As he states in an interview, ‘The training should be integrated in this therapeutic work, not the other way around. The first duty of the clinic is therapy, and when therapy is carried on all the time, the training can be integrated easily’ (Swerdloff, 2002, p. 390). Relying on some private subsidies from benefactors, the clinic offered psychoanalysis to those who could not afford it. It also paid the candidates who undertook clinical work at the clinic, which opened further possibilities to train psychoanalysts less able to sustain their training financially. On the whole, the work that went on at the Polyclinic showed great awareness of the social issues of the time.

In the midst of this dense psychoanalytic environment, Balint found the energies to reinitiate his project of reaching out to medical doctors and training them into psychoanalytic reflexivity. At the Polyclinic, he started a seminar for general medical practitioners. In this way, Balint was also hoping to enlarge the circle of psychoanalysts, by attracting new candidates (Hopkins, 1972, p. 317). Balint was still uncertain about the most suitable format for organizing this encounter between psychoanalysis and medicine. He reflected at a later point that the theoretical lectures he set up proved ‘fairly useless’ (Balint, 1970, p. 457). He had the intuition that the more productive approach would be to learn by practice and case presentations, and he experimented with a seminar dedicated to exploring the psychotherapeutic possibilities found in the everyday work of the medical doctors. All these observations would be extremely valuable at a later stage, in the 1950s, after his exile to the United Kingdom, when he would develop the practices around his ‘Balint groups’.

Around the same time, in 1930, Balint published the work ‘The Crisis of Medical Practice’ in the medical journal *Gyógyászat.* This text is strikingly ahead of its time, and it manages to articulate a critique of medicalization that maintains its relevance to this day. Here, Balint criticizes the fiction of the localization of the disease (*sedes morbi*), which assumes a pathological alteration in a particular function of the body. Thus, the task of the doctor becomes to discover and to attend to this impaired function (Balint, 2002, p. 9). As a result:

In the eyes of the doctor, the patient becomes an insensitive machine, a skilful combination of cleverly fitted parts; the totality of the person, a human being with his own goals and failures, his joys and sorrows, has practically vanished from their thinking. (Balint, 2002, p. 13)

It is here that Balint's ideas on ‘whole person medicine’ start to gain shape, to be later developed in his book *The Doctor, His Patient and the Illness* (Balint, 1964[1957]).

The exile to Manchester, from 1939 to 1945, brought loss and stasis. Michael lost Alice to a sudden death shortly after his arrival in the United Kingdom, and his letters of these years attest to the flatness of the period. In a letter to Judit Dupont-Dormandi, written on 21 February 1940, he speaks of his longing for the creative adventures in thought that he lived through with Alice:

I have many good relations, … but no friend as yet. Sometimes the loneliness falls on me so heavily that it is difficult to endure. You know, just these tiny half-born ideas one only can catch during a conversation, which we immediately presented to each other with Alice in order to get criticism, recognition or just encouragement, now these ideas just float around. (Dupont, 2002, pp. 361–2)

The metaphor of ‘floating’ marks in a strong way how Michael's state of feeling anchored occurred precisely when he was in conversation.

In the decade of the 1950s, with his move to London, Michael Balint entered his second creative dyad, that with Enid Eichholz, who would become his wife, his companion and his co-author. While their work together at the Family Discussion Bureau is often referred to as a key step in developing the method of the ‘Balint groups’, their theoretical co-authorship is less documented. Some of the ideas in *The Basic Fault*, signed by Michael in 1968 – especially those relating to the consequences of misrecognition – are prefigured in Enid's early work ‘On Being Empty of Oneself’, which appeared in 1963. In an interview collected by Peter Rudnytsky, Enid gives an account of the extent of the intellectual collaboration that resulted in writing *The Basic Fault*:

We discussed things. We wrote *The Basic Fault* together, but I didn't sign it. He wanted me to. Just before he died, I promised him that if there were a second edition, I would say Michael and Enid Balint. I never did, and I couldn't after he died. And I don't agree with it, though in fact I wrote quite a lot of it. All the bits around the malignant and benign forms of regression were mine, not his. That was my idea. (Rudnytsky, 2000, p. 14)

It is worth pondering why Michael would have left such a significant rectification to Enid, instead of co-signing the work with her in the first place. It might be precisely that in this case the form of the ‘individual author’ came to impose itself over that of the ‘dyad’, as the naturalized unit in the organization of knowledge. Perhaps Michael was not sufficiently reflexive about the politics of over-writing this collaboration. However, what is significant is that this dyad produced sufficient traces to allow for its reconstitution from written drafts, letters and interviews, instead of leaving a complete opacity around its specific creativities. It is also worth noting that Michael Balint was used to the presence of highly creative women psychoanalysts from his Budapest years. Balint experienced the strong presence of Vilma Kovács, his mother-in-law, who was leading the technique seminar at the Budapest Polyclinic. In an interview with Michelle Moreau-Ricaud, Judit Dupont-Dormandi clarifies that in the Kovács family it was women who were considered the most gifted ones (Moreau-Ricaud, 2000, p. 244). In the couple Alice–Michael Balint, it was Alice who was seen as one of the most promising voices of Hungarian psychoanalysis. Michael had to work to establish himself as her equal.

Balint's embeddedness in the Budapest School of psychoanalysis and the traces of his practices and collaborations, which we can gather from various writings and interviews, and from archival material, point to his multi-relational style and to his capacity for ‘making things’ (including psychoanalytic theory) in dyads or in collectives. The exchange with Eichholz detailed below is thus not a ‘curiosity’, but precisely a very condensed insight into Balint's epistemic style, which often involved a kind of back-and-forth between psychoanalysis and other fields of knowledge.

## Michael Balint and His Languages

In what follows, we unpack another crucial aspect of Michal Balint's epistemic style. His relation to different fields of knowledge and practices necessarily passed through his relationship with languages. The contours of Balint's ‘linguistic profile’ provide another frame for our analysis of the Balint–Eichholz correspondence.

Michael Balint was a polyglot. His mother tongue was Hungarian, while German was for him, as for any Hungarian intellectual in the first decades of the twentieth century, a kind of ‘father tongue’, the official language, the language of the Austro-Hungarian Empire. Pietr Judson (2016) has aptly shown how the Austro-Hungarian Empire creatively construed its unity across many divides of language, religion and custom. Balint himself would have felt this double belonging.

English was his language of exile, a language for the second part of his life. He also spoke French and had a good understanding of Latin, and some of Greek. There is something in Balint's linguistic portrait that points beyond his own intellectual gifts, and speaks about the beginnings of psychoanalysis. As Ferenc Erős (2016) has shown, psychoanalysis is a migration science, growing from and with the great dislocations of the first half of the twentieth century, with forced polyglots as much as with polyglots by vocation. An overwhelming number of psychoanalysts belonging to the first generations did not speak their mother tongue in their everyday life and in their clinical practice by the end of their lives. Furthermore, even if we think about the pre-Hitler times, psychoanalysts were often outsiders in their own countries (Erős, 2016; Jahoda, 1969), by functioning on the margins of the medical or of the university establishment.

In one of the boxes held at the Balint Archive, in London, a striking materiality records the major linguistic reinvention that Balint went through, after his move to the United Kingdom, in 1939. This box contains his patient diaries over 45 years. They are small objects, fitting comfortably in the palm of one's hand. Between 1926 and 1939, they are of Hungarian make. The only diary made in Germany is for the year 1925, marking his stay in Berlin. There are four missing years: 1940, 1941, 1942 and 1943. The years of the war. The years after the loss of Alice Balint. Perhaps this tells a story about how time itself died; or at least it became impossible to keep time, or to store the traces of its keeping. From 1944 until 1971, Balint kept his time in diaries of British make, of identical shape with the ones he was using in Hungary – on the first page we read: ‘John Walker and Co Ldt pocket diaries’.

In opening and closing the small diaries, one gets closer to Balint's journey across places and languages. When time was out of bounds, Balint preserved the shapes of his psychoanalytic work – just as he preserved the shape of his diaries. This brings us to one of Julia Kristeva's notes: ‘the only form of civilisation may be migration, a nomadism based on the strange ability some people possess of never identifying with “themselves” or “here” or “now”. The power to be always finding other places without losing their minds’ (Kristeva, 1994, pp. 149–50). Balint had this strange ability.

In a letter sent to Ladislas Dormandi, on 22 June 1961, after a trip to Budapest, Balint writes:

I could speak Hungarian – but still didn't feel at home. I knew every street, almost every house, and still was a foreigner. When finally we got to the English plane where a nice simple English steward welcomed us, I felt home at last. Who could understand that, but so it was. (Dupont, 2002, p. 378)

The letter, written in Hungarian, marks at the same time a painful falling-out of things-Hungarian. Still, the sense of homeliness is felt while on the plane, in-between spaces, rather than after landing in England.

In Balint's linguistic composition, German was the language of going away from Budapest, to Berlin, to study and to obtain his doctorate in natural sciences. But as noted above, it was still the language of a type of extended ‘within’ – the within of the Austro-Hungarian Empire, which gave him a feeling of familiarity. He could maintain a comfortable correspondence in German, but he did not feel at ease with speaking it freely, in front of psychoanalytic audiences. If invited to give talks in Germany, he would prepare a full written paper. His correspondence with Anna Freud is bilingual, moving from German to English, as both psychoanalysts settle more and more into their adoptive country.

Balint received letters in French, but he preferred to respond in English. His correspondence includes some lively exchanges with Jacques Lacan and Daniel Lagache, at the time they were breaking away from the French Society and thinking of a new organization. In these letters, Lacan chooses a cordial tone, he begins with the address ‘Cher ami’, and refers to the gross misunderstandings around the short sessions and their implications for psychoanalytic technique. Remarkably, in a letter written on 14 July 1953, Lacan makes an enigmatic remark, which contrasts with most of his comments on the work of the Budapest School: ‘dear friend, know that I always do a great part of my teachings in the lineage [*lignée spirituelle*] of Ferenczi […]’. Thus, in his rich correspondence, Balint forms numerous ‘occasional dyads’, which create a space for theoretical elucidations, confessions, critiques, clinical insights, and descriptions of the state of psychoanalysis or medicine in other countries.

The most complicated linguistic relationship is that with English, the language of Balint's adoptive country. His letters in English are elegant, playful and unconstrained. In his correspondence, we discover solid ‘silent dyads’, which although not visible in his published work, are crucial to one aspect or another of Balint's projects. Let us mention, for instance, the extensive letter exchange with Roger Francis Tredgold, based at University College Hospital, who was an anchor in Balint's work with medical doctors. The two dedicate hundreds of hours writing to each other, trying to get to the bottom of the various difficulties of principle and of planning that accompany any new endeavour relying on institutional support.

And yet, when Balint wishes to name two of his most important psychoanalytic discoveries, two kinds of object relationship, he is at a loss. The right word cannot be found. He turns to Latin and Greek and he initiates a naming-partnership with a classical languages scholar, David Eichholz. In this search for words, we can read the creative despair of the non-native speaker, who falls short of words. In his letters to Eichholz, when inventing composite words with Greek or Latin roots, Balint is worried about having created ‘monsters’. Are we here confronted with the dark side of the relationship of the polyglot to language, and with his fear that language might devour him or might even devour itself? Perhaps unknowingly, through his explorations in the Greek language, Balint comes closer to his Hungarian education. Greek has for him the valence of a secret mother tongue, rhythmic, but not yet broken into firm units. Balint writes to Eichholz, on 9 November 1953:

Despite my quoting Homer to you, my own [classical education] is not very deep […]. I owe my ability to quote to the fact that my Greek professor of anno dazumal used to insist that the best way of learning Greek was to learn long passages of Homer by heart. Although quite often I do not know what they mean, long pieces of Homer still jingle in my ears.

To the language ‘monsters’ of English, Balint prefers the ‘monsters’ of the Greek language.

## The Balint–Eichholz Correspondence

Between August 1953 and February 1954, Michael Balint and David Eichholz, an eminent classical languages scholar at the University of Bristol, exchange several letters in English, with a task in mind, which Balint himself proposes: naming two as yet unnamed object relations, which after this conversation will come to be known to psychoanalysts as ‘ocnophil’ and ‘philobat’. This is a passionate letter exchange, where the two protagonists labour at words, while they also free-associate in several languages to allow this creative labour to take place. David Eichholz's mind is used to dreaming in Greek, so as to touch the imaginaries of ancient philosophers. One of his papers, published in 1949, in *The Classical Quarterly*, is titled ‘Aristotle's Theory of the Formation of Metals and Minerals’, and it gives insights into Aristotle's ideas on states of aggregation. Balint lures Eichholz into the adventure of naming psychic states, while performing a kind of phenomenological seduction. He manages to describe to Eichholz the psychic ways he wishes to name, in an act of faith that the psychoanalytic objects at hand are indeed transmissible to other disciplines, that they can be understood by non-psychoanalysts, and that they can acquire a name through a conversation across fields of knowledge and across languages.

What is remarkable about this letter exchange is precisely Balint's phenomenological insistence, which both animates and irritates Eichholz. Balint's capacity for describing psychic states, in ways which make them readable to non-psychoanalysts, must have been influenced by Ferenczi, his mentor, and by Ferenczi's own habit of making such descriptions. A look at Ferenczi's papers on technique or at his *Clinical Diary* (Ferenczi, 1988[1932]) is sufficient to reveal the richness of the phenomenological detail, where entire paragraphs are dedicated to answering questions such as: what happens to the psyche at the time of trauma? What becomes of the split-off parts of the psyche after the traumatic moment? Out of Freud's writings, it is perhaps only in ‘Mourning and Melancholia’ that we find such phenomenological satisfactions.

This letter exchange is also the place for a clash between two different conceptions of language: Balint's associationism and his non-arbitrary theory of language (where, as we will see, Ferenczi's ideas on mimesis play an important part) meets Eichholz's arbitrary theory of language. Eichholz is initially perplexed at Balint's insistence on the presence of a ‘halo of associations’ around each word, he is then seduced by the associationist proposition, and he plays the Ancient Greek imagination game that Balint invited him to.

Balint was influenced by Ferenczi's conception of language, and by his sophisticated idea of mimesis between words and things. Language is both physical and psychic. There is an inscription of materiality at the core of every word. This inscription results from the operation of analogy, through which symbols are made. The primary analogies take us back to the body and to the child's act of establishing correspondences between body parts and external reality. Just as symbols express the body, words imitate things. As Ferenczi writes: ‘In its origin, language is imitation, in other words, vocal reproduction of sounds and noises produced by things, or that are produced through them’ (Ferenczi, 1913, p. 228). And, later in his *Clinical Diary*, he adds: ‘To speak is to imitate. The gesture and speech (voice) imitate objects of the world around. “Ma-ma” is magic of imitation’ (Ferenczi, 1988[1932], p. 151).

The halo of associations that surrounds every word is both material and sensorial. This is why Balint had the habit of ‘dreaming up’ his theoretical terms and following the trails of associations that each word invited him to take. In a letter to James Strachey, where Balint voices his critique of Strachey's neologistic and over-scientific choice of words in his translation of Freud, we see where his heart lies when it comes to the naming of psychoanalytic objects:

This leads me to a very difficult problem which may be termed the relationship between language and depth-psychology. My problem is to decide what the advantages and disadvantages are of using a precise unequivocal term for a complex and over-determined observation or inference, as compared with using an everyday unprecise word. A very good example of this is the German ‘Besetzung’ and the English ‘cathexis’. The first is highly unprecise but stimulating; for instance, in my case I associate with it a small gallant corps defending a fortress against the onslaught of the enemy and turning their guns outward, and, parallel with it, an Army turned inwards and oppressing the population say, like the Germans did with the French, or the Russians are doing now with Hungary. In contrast, the exact English word ‘cathexis’ does not stimulate me at all.

Most of Freud's technical terms belong to the first category. They are over-determined, and most of them are inexact, but they stimulate one's phantasy and thus press for experiencing and interpreting them simultaneously at different levels. (Letter of Michael Balint to James Strachey, 25 August 1959)

Given this love of everyday words, why would Balint have gone in search of Ancient Greek roots for his terminological inventions? This has to do with his relationship to the English language, and his suspicion that he could not ‘dream up’ an English word to match his phenomenological descriptions. An abstract of a lecture that Balint gave at the Department of Philosophy of Smith College, in Northampton, Massachusetts, in 1966, holds some clues as to why Balint summoned David Eichholz to help him find the right words.^5^ The abstract reads:

My idea is that man apparently cannot stand exactly defined concepts or ideas, although beautiful, they are alien to his nature. As soon as he purifies and clarifies any one of them he must start to extend, to twist them or make them hazy, with uncertain boundaries. All these ‘machinations’ are reflections of his unconscious wishes and conflicts, and obey much more the rule of the original primary processes than the later, much more secondary processes. What is created by these ‘machinations’ is what I called the cluster of associations which surrounds practically every word in every language.

Balint must have felt that for the task at hand, he was incapable of the necessary ‘machinations’ in English, and thus devised a new context for naming: a trans-linguistic dyad, which would produce a Greek solution – a language that, as noted previously, was for Balint linked with primary process: it was rhythmic, hazy, experienced as a flux rather than as broken into discriminate units.

In what follows, I cite extensively from the correspondence, rather than using small excerpts as illustrations to support a single argument. This is because we are discussing a linguistic quest, and the twists and turns of language, as well as the chains of synonyms, and, indeed, the associations surrounding them in the same paragraph are likely to be of great interest to readers. This correspondence is also new to the psychoanalytic community, which makes the exercise of extensive citations even more important. Let us see how Balint introduces his search for words to Eichholz:

I am still labouring with my Fun Fair, and come to you, the erudite and classical scholar, for some help with words. Latin words would do, but if you can provide Greek ones I would much prefer them. Moreover, I need some very flexible ones with which one can play in order to express the various connotations, as for instance, (a) sadism –ist –istic, or, still better (b) ideal –ist –ism –ise –isation, – it is a great pity that ‘genitalist’ cannot be formed.

I have two ideas to describe and for each of them I would need a couple of words which are the opposite of each other.

1(a) Will you bear in mind the raving madman's kitchen in a funfair. What I need is an adjective to describe the instinct for and the pleasure in breaking up things in the external world, a noun for the action itself and an abstract noun to denote the whole field.

Something like iconoclast would be alright, then you would have iconoclastic or iconoclasis. It would be still better if the word would lend itself to such derivatives as iconoclasticise and iconoclasticism.

1(b) The same requirement for another word to describe the opposite propensities, i.e. preserving the external world whole and safe.

2(a) Will you bear in mind now the people who go on a switchback, but have to hold on desperately to some bar or something in order to bear the tension, and those who do not even dare to leave the safe earth and who consider any such thrill almost a mortal danger. There are a whole cluster of English verbs to describe this kind of internal urge, such as cling, clutch, stick, grasp, stay, remain, but no one of them lends itself to be changed according to the many uses for which I need it. Moreover, there is a ‘cluster of associations’ round each of them, which would lead the reader to imagine quite different things from where I want to take him. For instance, if I call someone a ‘clutchist’ or a ‘clutcher’, everybody would think of some neurosis connected with the motorcars. The same is true of ‘grasper’ or ‘sticker’ or ‘clinger’; the associations do not lead in the direction I require.

The interesting thing is that in Latin the corresponding word to grasp has almost the associations that I look for, comprehend, apprehend, prehensile, prehensive, and so on, and it is very nice the old Romans thought ‘apprehensive’ means rather than timid, someone whom we can almost see clutching desperately to something to keep up his courage. I experimented with the word ‘statophilic’ but it is no good whatsoever.

2(b) The opposite word should denote the urge to let something go, to look or search for something, to move away from something. Here too I experimented with the monster ‘motophilic’, but again I found that most people thought of somebody who is a mad motorist, which is not too bad, but not quite the right thing.

As far as I remember there was a Greek demi-god, Gaia's son, who was strong as long as he could remain in touch with his mother, and even Hercules could not kill him unless he held him away from his mother in the air. Perhaps you could suggest some word derived from his name, like sadist from the Marquis de Sade.

As you see, I only ask for four words, which is not much on this face value, but I know from experience how very difficult it is to find the right one, and I would be most grateful for help or suggestions. (Letter of Michael Balint to David Eichholz, 10 August 1953)

To this first exposition, where Balint introduces his associationist theory of language, and he expresses his fears of language ‘monsters’, Eichholz replies with some reserve. More phenomenological stubbornness will be required from Balint to fully engage the classical scholar:

1a. Of course I know the madman's kitchen. It was always my favourite sideshow. The only pity was that they were comparatively very rare. What about sceuoclastic, i.e. breaking vessels or implements? This may be too restricted: unfortunately there is no Greek noun for the external world as such. One would have to use the definite article with an adverb, and this makes compounds impossible.

2a. This beats me. Topophilic? Or with the emphasis on ‘grasping’, sylleptic?

2b. Possibly planophillic, i.e. liking to wander. Or if you emphasise ‘letting go’, aphetic (which is the only one of these suggestions I like).

The giant who was thrown by Hercules was Antaeus. He would give you Antaean and, if you could bear it, anti-Antaean. (Letter of David Eichholz to Michael Balint, 20 August 1953)

The traces of this conversation are marked by Balint (1959) in two footnotes in his book, *Thrills and Regressions.*^6^ Balint writes in the first footnote: ‘I wish to express my gratitude to David Eichholz, Reader in Classics at the University of Bristol, who, greatly amused by my efforts to find suitable words for my ideas, helped me to devise these two terms’ (Balint, 1959, p. 25). Balint's acknowledgement makes the encounter appear as a kind of jovial word-play. In fact, Eichholz's letters do not read as ‘amused’; they are engaged, imaginative, committed to labouring at words, as well as irritated and ironic at Balint's particular views on the ways in which language works.

The two terms that were born in this conversation, ‘ocnophil’ and ‘philobat’, occupy an important place among Balint's theoretical innovations. In *The Basic Fault*, Balint (1968) discusses how in the moment of the birth trauma, there are changes that occur in the ways the libido encounters the environment. In this moment, objects (including the ego) begin to emerge as sharper and with more contour, from a previously harmonious mix-up of substances. As he writes, ‘Libido is no longer in a homogenous flux from the id to the environment; under the influence of the emerging objects, concentrations and rarefactions appear in its flow’ (Balint, 1968, p. 67). Balint believes that the narcissistic libido, whose cathexis is the developing ego, is secondary to the original environment cathexis. For him, there are four kind of cathexes observed in early childhood: (a) remnants of the environment cathexis transferred to the emerging objects; (b) other remnants of the original environment cathexis withdrawn to the ego as secondary comforters against frustration, i.e. narcissistic and auto-erotic cathexes; (c) re-cathexes emanating from the secondary narcissism of the ego; and (d) a kind of cathexis that results from the ocnophilic and philobatic structures of the world – it is here that Balint makes his contribution (Balint, 1968, pp. 67–8). As he explains, in the ocnophilic world of primary cathexes, emerging objects are experienced as safe and comforting, while the spaces between them are threatening and horrid. In the philobatic world of primary cathexes, it is objectless cathexes that are experienced as safe and friendly, meanwhile objects are felt as treacherous hazards. The ocnophil meets the emergence of objects with a tendency to cling to them, to introject them, or to over-cathect his object relationships (Balint, 1968, p. 68). The philobat, by contrast, over-cathects his own ego-functions.

Faced with Eichholz's reserved answer, Balint details the chains of associations he has in mind, and he spells out some of the resonances contained in the halo of each word proposed, which would make it an unhappy solution for his metapsychological construction:

1 a and b. Sceuo-clastic-philic. The pronunciation suggested by you, i.e. rhyming with Kew, will raise the association of ‘skewy’, which is certainly one that I would like to avoid. Moreover, vessels or implements are rather narrow; I would like to have some quite general word like ‘object’ is in Latin. May I suggest two directions where perhaps something might be found? One is in the Greek word for the grammatical object – I do not know it, but I suggest that as Aristotle wrote grammar he very likely made some distinction between transitive and intransitive verbs, and very likely arrived at the subject of a grammatical object. The other – of this I have some vague memory – going back also to Aristotle, who had some Greek word for what the Germans called ‘Ding an sich’, in contradistinction, again if I remember correctly, to Plato's ‘idea’. My main aim is to describe something real, which can be either preserved or destroyed. […]

2 b. ‘Planophilic’ again leads to the association of plane in a geometrical sense, or aeroplane in the mechanical sense, which is not the direction I would welcome. Letting go, aphetic I agree is the best; the trouble is that it does not lead to any association, at all, because nobody knows this word, and I do not know whether it is flexible enough, for instance, to change it to ‘aphetism’ and ‘aphetical’ and so on. From my old Greek days Odysseus comes to mind, who was a wanderer indeed. I think one of his constant epithets was ‘polytropos’ which perhaps could be used as ‘polytropic’ and ‘polytropism’, possibly even ‘polytropical’. I am certain he had other epithets which possibly might be of some use. (Letter of Michael Balint to David Eichholz, 22 September 1953)

In response to this detailed play on the halo of words, Eichholz brings the conversation to a double climax: he is irritated by the implications of Balint's theory of language for naming psychic states and confronts him with the radicality of his associationism; but he also makes the most productive linguistic suggestions, which Balint will embrace thereafter:

Now as for your word-making, I must confess that your specifications seem to me to be altogether much too rigorous. How can any words stand up to the tests which appear to presuppose that a word can be made to inhabit an ideal world of strict orthodoxy, in which none of its associations will be ‘trefa’ and all will be ‘kosher’? And some of the associations which you find, and find objectionable, are really too preposterous. One would almost think that you give your articles to your patients to read as material for free association, in which case I can't see that it matters what words you use. But if your readers are intelligent people who, when they are reading a scientific exposition, are able to control their thoughts and resist stock-response, then you ought to be able to take calculated risks with your vocabulary, since your public should be able to deduct without difficulty any irrelevant associations that may present themselves. I admit that in a poem it is often hard to do this, but you are writing an article not a lyric. At least, I hope you are. Having worked this off my chest, I can get down to business.

[…]

I hope that the new words are a slight improvement on the old. If you do approve of ‘ocnobatic’ and ‘philobatic’, I am sure that you will do so for the wrong reason, that is, because they remind you of ‘acrobatic’. (Letter of David Eichholz to Michael Balint, 23 September 1953)

In the next exchange, Balint insists that ‘stock-response’ is impossible to avoid, as readers cannot control their thoughts across chains of associations. He also becomes very interested in the terms proposed by Eichholz, and asks additional questions about their roots. Eichholz gives a bifurcated answer once again: on the one hand, he engages Balint's associative play; on the other hand, he ironically gives Balint a ‘diagnosis’ for his relentless search for the right word:

‘Ocno-’ is from ‘oknos’, hesitation, reluctance, and the verb ‘oknein’, to hesitate, etc. ‘-batic’ is the same root as ‘bainein’ to step, go, move. An Acrobat is literally one who steps on tiptoe. ‘Ocnobatic’ and ‘philobatic’ are again my own invention. They mean hesitating, fearing to go, and liking to go, move. ‘Oknos’ includes hesitation, reluctance, sluggishness, timidity, alarm. As for ‘philo-’ I need not tell you all what that can mean.

‘To stick, cling’: glichesthai – no good for compounding. ‘To be afraid to move’: my word ‘ocnobatic’ should cover this. ‘To turn away’: I offer my own coinage ‘apotropic’. Another possibility is ‘apostrophic’, but this is, to my way of thinking, too close to ‘apostrophize’, ‘apostrophe’. ‘Stand alone’: possibly ‘monostatic’, another of my inventions. Or does this remind you too much of Monostatos in the Magic Flute? ‘Intent on looking about’: I am tempted to invent ‘perisceptic’. What about the association with ‘sceptic’? ‘To search around’: ‘diazetetic’ or ‘diereunetic’ are both ugly. But Lindell & Scott, who rarely find Latin equivalents actually give ‘rerum novarum studium’ as the equivalent of ‘neoterismos’ (where the first ‘o’ is long). Literally this means ‘going in for something newer’, i.e. ‘making innovations’. From this you could form ‘neoterist', neoterism’, ‘neoteristic’.

[…]

Entertaining as this is, I do feel that I'm getting out of my depth and I only hope that I've provided something that will work. Having invented so many words, may I be allowed to coin one more and suggest tactfully and kindly that you show certain, unmistakable symptoms of Adynatomania? By which I mean a craze for the impossible. It could also mean impotent madness, which is what I'm suffering from at the moment. (Letter of David Eichholz to Michael Balint, 1 October 1953)

Balint accepts Eichholz's diagnosis – adynatomania – but the play on words continues, as he takes this new invention for proof that his own associationist theory of language is correct. He argues that a search for a word that contains a reference to both subject and object is able to call forth in the mind of the interlocutor precisely a term with this double subject–object reference – even if this is presented in the form of a friendly-irritated diagnosis for a pathological relation to language:

In spite of the exasperation and irritation caused […], your last paragraph shows that I have succeeded in making you see what the problem is. By this I refer to your newly coined word, when you suggest ‘tactfully but kindly’ that my behaviour shows unmistakable signs of ‘adynatomania’, meaning a craze for the impossible. You add immediately, in the next sentence, ‘it could also mean impotent madness’, which is what you are suffering at the moment. You could not have given a more eloquent proof of the importance of what I call ‘cluster of associations’. Adynatomania is an excellent word to describe at one and the same time the behaviour of the subject, and the effect of that behaviour on the object … There are very few words that could be used to describe a relation of this kind between two people, and this sort of word is the one I was looking for to describe other kinds of relations between two people. Thank you very much for the many excellent suggestions in your letter. I do not want to hurry now with any praise or criticism; I wish to live with them for some time and to find out how they feel, and which of them is a handy and useful tool and which needs improvement. (Letter of Michael Balint to David Eichholz, 7 October 1953)

Balint thus marks that their encounter happens in a relational space, in a pre-existing tangle of words and in a pre-existing tangle of words and things, where some threads call for others. The main and irreducible encounter is that between subject and object, but neither subject nor object is fixed in position, rather they take each other's places. The existence of this letter exchange itself stands for this change in positions. Balint's response is an epistemologically complicated one, under the appearance of witty playfulness. It suggests to us a continuous oscillation between one and the other, between introjection and projection. What Balint suggests is that both the pair ‘ocnophil’/‘philobat’ and Eichholz's invented diagnostic category ‘adynatomania’ inhabit the same ontological space, where what is presupposed is precisely the relation, the encounter between subject and object.

Once the words have been coined, Balint announces that he intends to live with them, so as to see how they feel in relation to the psychic states he wishes to describe. The dyad seems here to turn into a triad: it is Balint and Eichholz and the words they created together. In a letter written on 30 October 1953, Balint reiterates: ‘As promised I have been living with your proposed words and find some of them quite good companions.’ After 1953, many psychoanalysts will have lived in the company of these words.

## Conclusions

One of the most remarkable aspects of the Balint–Eichholz correspondence is the encounter between two different theories of language (one that we could describe as ‘non-arbitrary’ and the other as ‘arbitrary’), not just between different languages. We followed above every step of a passionate exchange, which does not lead to perplexity or to the interruption of communication. On the contrary, Balint makes all possible efforts to describe to Eichholz how language works for psychoanalysts, and how he thinks that even theoretical concepts bear a halo of associations, which makes the choice of the concept an important part of the theoretical exercise. The psychoanalyst will thus not cease to ‘dream up’ their concepts. In response, Eichholz lets his linguistic imagination run free and together they settle for ‘ocnophil’ and ‘philobat’. As I suggested, this mode of ‘making things together’ is not an isolated event in Balint's trajectory, but can be read through the frame of the practices of the Budapest School of psychoanalysis. One of Balint's most creative modes of functioning was as part of a dyad. This insistence on dyads and on other collective modes of producing knowledge (including his experimentations with ‘Balint groups’) confronts us with an important epistemological challenge, and leads us away from putting the individual author at the centre of our preoccupations. Balint's capacity to function in creative dyads is also bound with broader and equally relevant conversations about the collective practices of the first generations of psychoanalysts, who led complicated collective lives, being tied together in multidisciplinary groups or in social clinics.

## Notes

1. It is worth noting that several voices have made significant contributions to understanding the trauma history of psychoanalysis. See Steiner (2000); Zaretsky (2005); Burnham (2012); ffytche & Pick (2016). These histories, however, have not been crystallized into a widely accepted self-understanding of the psychoanalytic field, one that can inform the everyday lives of psychoanalytic institutes and their organization of psychoanalytic training and transmission.

2. In *The Basic Fault*, Michael Balint himself spoke of the magnitude of the consequences of the split between Freud and Ferenczi: ‘The historic event of the disagreement between Freud and Ferenczi […] acted as a trauma on the psychoanalytic world’ (Balint, 1968, p. 152).

3. The letters are part of the Balint Archive, held by the British Psychoanalytical Society, in London. It was previously held by psychoanalyst and historian André Haynal, in Geneva.

4. In a personal communication, in December 2018, Susan Lawlor, Enid Balint's granddaughter, clarified that David Eichholz was one of the brothers of Enid's first husband.

5. The lecture was not preserved. The abstract is held by the Balint Archive.

6. A previous work discussing the same topic is ‘Friendly Expanses – Horrid Empty Spaces’ (Balint, 1955).
